# Distributed and Scalable Radio Resource Management for mmWave V2V Relays towards Safe Automated Driving

**DOI:** 10.3390/s22010093

**Published:** 2021-12-23

**Authors:** Yue Yin, Tao Yu, Kazuki Maruta, Kei Sakaguchi

**Affiliations:** 1 Department of Electrical & Electronic Engineering, Tokyo Institute of Technology, Tokyo 152-8550, Japan; yutao@mobile.ee.titech.ac.jp (T.Y.); sakaguchi@mobile.ee.titech.ac.jp (K.S.); 2 Academy for Super Smart Society, Tokyo Institute of Technology, Tokyo 152-8550, Japan; maruta.k.aa@m.titech.ac.jp

**Keywords:** mmWave V2V with relays, required data rate, distributed radio resource management, scalable

## Abstract

The millimeter-wave (mmWave) Vehicle-to-Vehicle (V2V) communication system has drawn attention as a critical technology to extend the restricted perception of onboard sensors and upgrade the level of vehicular safety that requires a high data rate. However, co-channel inter-link interference presents significant challenges for scalable V2V communications. To overcome such limitations, this paper firstly analyzes the required data rate ensuring maneuver safety via mmWave V2V relays in an overtaking traffic scenario. Based on these preparations, we propose a distributed radio resource management scheme that integrates spatial, frequency, and power domains for two transmission ranges (short/long). In the spatial domain, ZigZag antenna configuration is utilized to mitigate the interference, which plays a decisive role in the short inter-vehicle distance. In frequency and power domains, two resource blocks are allocated alternately, and transmit power is controlled to suppress the interference, which has a decisive impact on interference mitigation in the long inter-vehicle distance. Simulation results reveal that the achievable End-to-End (E2E) throughput maintains consistently higher than the required data rate for all vehicles. Most importantly, it works effectively in scalable mmWave V2V topology.

## 1. Introduction

Vehicle-to-Vehicle (V2V) communication has been identified as an essential technology to improve driving behaviors and increase safety levels of autonomous vehicles since it can unleash the restriction of onboard sensors, break the line-of-sight constraints, and enhance the overall contextual awareness by acquiring more data from surroundings. To support advanced V2V use cases [1,2], exchanging raw sensor data is necessary, which poses demanding requirements on V2V [3]. For instance, a data rate over 1 Gbps and End-to-End (E2E) latency of less than 10 ms per link is the typical requirement for extended sensors [4]. Having large continuous spectrum resources, millimeter-wave (mmWave) becomes a promising frequency band to support such high data rate V2V communications. For example, the IEEE 802.11bd [5] is being specified to adopt to advanced V2X (Vehicle-to-Everything) applications, assuring backward compatibility with IEEE 802.11p [6] and IEEE 802.11ad [7]. Its peak data rate will be higher than 6.75 Gbps. New Radio V2X (NR-V2X) [8,9] is also being specified by 3GPP (3rd-Generation Partnership Project) with a peak data rate of 20 Gbps.

Wireless communication in the mmWave band experiences a higher signal attenuation and blockage than conventional frequency bands such as 760 MHz and 5.9 GHz. Although high-gain directional antennas and multi-hop relaying are employed to cope with the blockage, many other challenges remain, such as the scalability of V2V topology and interference management. To the best of our knowledge, most related works about mmWave radio resource management are so far limited to beam allocation and alignment [10,11,12]. All of them study single-hop mmWave V2V link scenarios. We previously proposed a ZigZag antenna configuration to mitigate the interference among mmWave V2V relay links [13]. Although it can maintain a throughput of over 1 Gbps by reusing a single channel, this spatial resource control was only suitable for two mmWave V2V links and short inter-vehicle distance. This has become one of the critical issues that need to overcome in order to design scalable radio resource management for mmWave V2V relays.

In addition, a dynamic V2V topology formed by high mobility vehicles requires a fast resource management mechanism. It can be divided into two types: centralized and distributed radio resource controls. The centralized one is more reliable. For instance, Mei et al. and Ashraf et al. proposed the centralized resource allocation schemes for Long Term Evolution V2V (LTE-V2V) communication systems to guarantee reliability requirement [14,15]. Gao et al. targeted the energy efficiency and proposed a centralized power control and resource allocation for V2V communication [16]. These centralized radio resource management schemes have high control latency, high uplink transmission cost, and cannot work out of coverage of the central node. By contrast, distributed radio resource control can reduce the control latency and work even out of coverage. However, existing distributed schemes mainly adopt IEEE 802.11p as an access layer, which cannot support services that require a data rate over 1 Gbps [17,18]. Crucially, the collision of resource utilization becomes inevitable as the traditional Carrier Sense Multiple Access with Collision Avoidance (CSMA/CA) strategy suffers from its low efficiency. Therefore, the design of distributed radio resource mechanisms in dynamic vehicular scenarios to reduce the control latency and avoid the collision of resource utilization is another essential issue.

To cope with the above two issues, a distributed radio resource management scheme is proposed for a scalable mmWave V2V relay topology to cope with the above two issues. Our proposal aims to ensure that the achievable E2E throughput satisfies the required data rate for all vehicles in the overtaking scenario. It is noteworthy that, in this proposal, individual nodes have autonomy in resource management rather than relying on the global knowledge from a centralized control node, rapidly adapting to the dynamics of mmWave V2V communications.

The main contributions of this work are outlined as follows:

(1) The required data rate considering driving safety is analyzed in mmWave V2V communication with relays at different inter-vehicle distances and vehicle speeds in an overtaking traffic situation, which is the basis for radio resource management;
(2) A distributed radio resource management scheme is proposed to ensure mmWave V2V relaying topology scalability; First, ZigZag antenna configuration is employed in the spatial domain to mitigate the inter-link interference impact caused by the reuse of resource blocks (RBs). Further, in frequency and power domains, two transmission modes (Mode 1 and Mode 2) are defined according to the inter-vehicle distance and are switched based on the required data rate for each V2V link. The full available bandwidth is divided into two RBs, called $\{{\mathrm{RB}}_{1}$, ${\mathrm{RB}}_{2}\}$, respectively. In mode 1, all bandwidth is used for the current V2V link. In mode 2, one of ${\mathrm{RB}}_{1}$ and ${\mathrm{RB}}_{2}$ is selected for the current V2V link. If continuous V2V links are in the same mode, ${\mathrm{RB}}_{1}$ and ${\mathrm{RB}}_{2}$ are alternately allocated for each V2V link in a ZigZag manner.

Theoretical analysis and simulation verify the effectiveness of the proposed scheme, which can guarantee achievable E2E throughput higher than the required data rate for all vehicles in an overtaking scenario.

The remainder of the paper is organized as follows. Section 2 analyzes the required data rate for mmWave V2V relay communication; Section 3 explains the proposed distributed resource management scheme in detail; Section 4 presents the simulation results. The conclusion is drawn in Section 5.

## 2. Required Data Rate for Extended Sensors in Overtaking Scenarios

This section defines the overtaking scenario with multiple Ego vehicles and theoretically analyzes the required data rate for safe automated driving. Then, the supposed V2V relay scheme is described where rate control (down sampling) is introduced as a mechanism to match the data rate with corresponding requirements during multi-hop relay. Finally, some numerical examples reveal the required data rate with different speeds of the Ego vehicle and inter-vehicle distances.

### 2.1. Overtaking Scenarios

**Figure 1:**
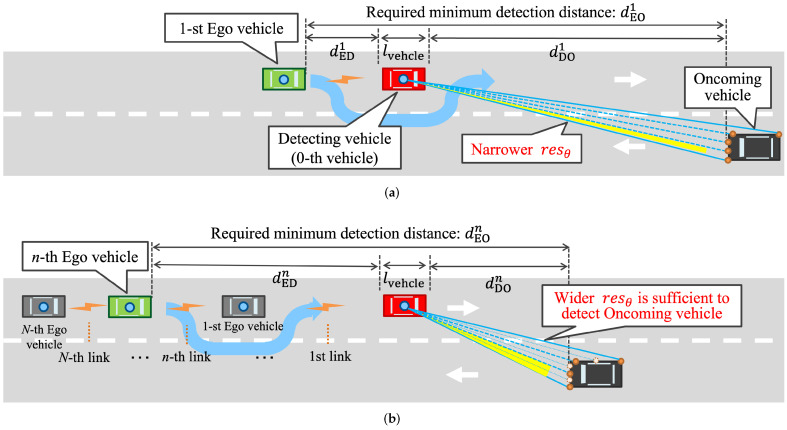
MmWave V2V relays with extended sensors in overtaking traffic situation. (**a**) Single-hop case: Oncoming vehicle is far from Detecting vehicle, which requires a higher data rate for LiDAR with narrower (original) resolution. (**b**) Multi-hop case: the oncoming vehicle is closer to the Detecting vehicle where a lower data rate is sufficient for LiDAR with wider (down sampled) resolution.

Although the original study of safe overtaking by cooperative perception was introduced in [3], it only analyzed a fundamental setup of cooperative perception in the case with a single Ego vehicle and a single V2V link as shown in Figure 1a. In practical traffic scenarios, there are usually more than two vehicles. This paper bridges this gap by extending the situation to multiple Ego vehicles and multiple V2V communication links, as shown in Figure 1b. This paper considers the overtaking traffic situation in a straight two-lane road with width $w_{\mathrm{lane}}$. N+1 vehicles, equipped with a light detection and ranging (LiDAR), which are running in the middle of their lanes. Let the 0-th vehicle be the Detecting vehicle and following *n*-th (n∈{1,⋯,N}) Ego vehicles are trying to overtake its front vehicle safely by avoiding collision with the Oncoming vehicle. Since the perception of each Ego vehicle is blocked by its front vehicles, the Ego vehicle requests the LiDAR data on the Detecting vehicle to perform cooperative perception. The rate of exchanging raw sensor data for safe overtaking is defined as the required data rate.

To achieve safe overtaking, the *n*-th Ego vehicle must detect the Oncoming vehicle at the minimum distance from itself. Otherwise, the Ego vehicle will collide with the Oncoming vehicle during overtaking. Here, this minimum distance is called the required minimum detection distance $d_{\mathrm{EO}}^{n}$, as shown in Figure 1. This required minimum detection distance is defined as the sum of the individual required braking distances of the Ego vehicle (*n*-th vehicle) and Oncoming vehicle for collision avoidance in an overtaking scenario such as $d_{\mathrm{EO}}^{n}=0.039\times \frac{{v_{e}^{n}}^{2}+{v_{o}}^{2}}{3.4}$ [3], where $v_{e}^{n}$ and $v_{o}$ are the speeds of *n*-th Ego vehicle and Oncoming vehicle, respectively. It shows that $v_{e}^{n}$ and $v_{o}$ are the hidden parameters that determine the required $d_{\mathrm{EO}}^{n}$. This study assumes that all Ego vehicles and the Oncoming vehicle are traveling at the same speed ($v_{o}=v_{e}^{n}=v_{e},n\in \left\{1,\cdots ,N\right\}$), so the required minimum detection distances of all Ego vehicles are same $d_{\mathrm{EO}}^{n}=d_{\mathrm{EO}},n\in \left\{1,\cdots ,N\right\}$.

### 2.2. Required Data Rate for Extended Sensors

Figure 1 presents simplified examples of V2V relay-based Oncoming vehicle detection to provide an intuitive understanding of required data rates. In the single-hop case of Figure 1a, i.e., n=1, the first Ego vehicle can detect the Oncoming vehicle with the assistance of the front Detecting vehicle. Multi-hop case is shown in Figure 1b. In front of the *n*-th Ego vehicle, several relay vehicles follow the Detecting vehicle. Under the premise of equal required minimum detection distance $d_{\mathrm{EO}}^{n}$, the Detecting vehicle is relatively close to the Oncoming vehicle compared with the single-hop case. Hence, LiDAR can recognize it even with wider angular resolution; point cloud data can be down sampled, reducing the required data rate. To rephrase, the required data rate is inversely proportional to the Ego-Detecting vehicle distance, $d_{\mathrm{ED}}^{n}$. Its detailed definition is described below.

**Figure 2:**
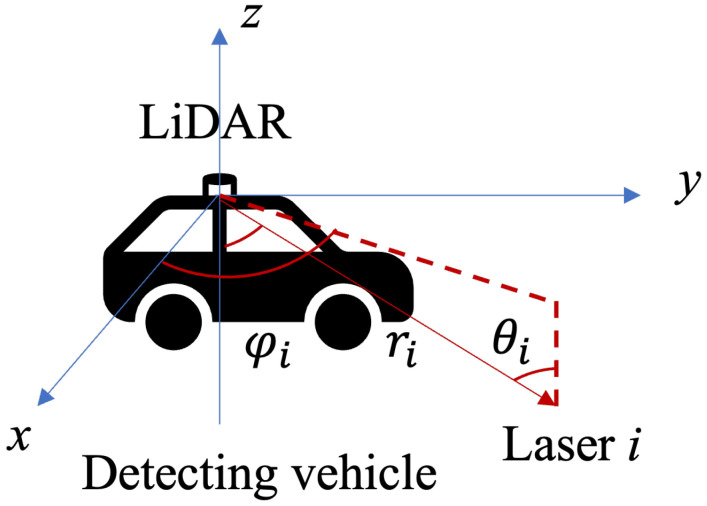
The coordinate of LiDAR on the Detecting vehicle.

**Figure 3:**
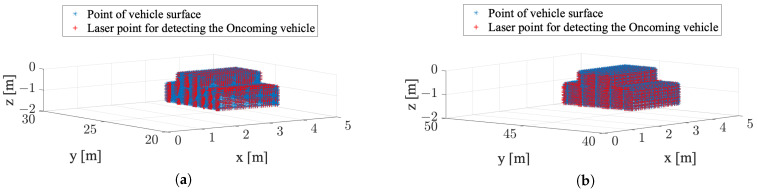
Model for detecting Oncoming vehicle by LiDAR on the the Detecting vehicle. (**a**) $S_{n}\left(d_{\mathrm{DO}}^{n}=20\;m\right)\geq 90\%$; (**b**) $S_{n}\left(d_{\mathrm{DO}}^{n}=40\;m\right)\geq 90\%$.

A coordinate system is established with the position of LiDAR on the Detecting vehicle as to the origin, as shown in Figure 2. The position of each laser point (i.e., laser *i*) can be expressed as $r_{i}\times \left[\sin\theta_{i}\cos\phi_{i},\sin\theta_{i}\sin\phi_{i},\cos\theta_{i}\right]$, where *i* is the index of the laser beam, $r_{i}$ is the distance between the origin and laser point on the obstacles, $\theta_{i}$ is the angle between the laser beam and the negative z-axis, $\phi_{i}$ is the angle between the laser beam and the positive x-axis. Therefore, the geometric model of LiDAR can be created as shown in Figure 3. The point cloud model of the vehicle’s surface is then imported into the coordinate system. In this figure, (0,0,0) is the position of LiDAR on the Detecting vehicle. The blue points represent the vehicle model of the Oncoming vehicle, and the red points represent the laser points from LiDAR detecting the Oncoming vehicle. In the scenario of this paper, only the side surface near the LiDAR, the roof, and the front surface of the Oncoming vehicle can be detected by the Detecting vehicle; only these three surfaces are taken into consideration. Let the number of detected points by the LiDAR denote $N_{\mathrm{detected}}$, and the total number of points of vehicle model is $N_{\mathrm{vehicle}}$. Their relationship $S_{n}$, is expressed as

(1) $$S_{n}=\frac{N_{\mathrm{detected}}\left(d_{\mathrm{DO}}^{n},res_{\theta },res_{\phi }\right)}{N_{\mathrm{vehicle}}},$$

where $d_{\mathrm{DO}}^{n}=d_{\mathrm{EO}}^{n}-d_{\mathrm{ED}}^{n}-l_{\mathrm{vehicle}}$ in Figure 1. Based on the assumptions in Section 2.1, $v_{e}^{n}$ is the hidden parameter that determines the required $d_{\mathrm{EO}}^{n}$. $d_{\mathrm{DO}}^{n}$ can be substituted with $d_{\mathrm{ED}}^{n}$ and $v_{e}^{n}$ in Equation (1), which is rewritten as

(2) $$S_{n}=\frac{N_{\mathrm{detected}}\left(d_{\mathrm{ED}}^{n},v_{e}^{n},res_{\theta },res_{\phi }\right)}{N_{\mathrm{vehicle}}},$$

where $res_{\theta }$ and $res_{\phi }$ are the angular resolution in vertical and horizontal planes, respectively. When $v_{e}^{n}$ and $d_{\mathrm{ED}}^{n}$ are given, $N_{\mathrm{detected}}$ can be changed by adjusting the angular resolution $res_{\theta }$ and $res_{\phi }$. Since $N_{\mathrm{vehicle}}$ is fixed, $P_{n}$ can be changed by adjusting the angular resolution $res_{\theta }$ and $res_{\phi }$. This paper assumes that LiDAR on the Detecting vehicle can detect and identify the Oncoming vehicle when $S_{n}\geq 90\%$. Meanwhile, $res_{\theta }^{n}$ and $res_{\phi }^{n}$ are recorded and used to calculate the required data rate of *n*-th vehicle according to Equations (3) and (4).

(3) $$\left\{res_{\theta }^{n},res_{\phi }^{n}\right\}=\arg\min S_{n}\left(res_{\theta },res_{\phi }|v_{e}^{n},d_{\mathrm{ED}}^{n}\right)\geq 0.9$$

(4) $$R_{\mathrm{req}}^{n}=f_{\mathrm{scan}}B_{\mathrm{laser}}\left(\left\lfloor \frac{\Theta }{res_{\theta }^{n}}\right\rfloor +1\right)\left(\left\lfloor \frac{\Phi }{res_{\phi }^{n}}\right\rfloor +1\right)$$

where $f_{\mathrm{scan}}$ is the scan frequency of LiDAR, $B_{\mathrm{laser}}$ is the number of bits per laser, Θ and Φ are the angular scan range in horizontal and vertical planes, respectively.

### 2.3. V2V Relay Communications

In the topology of V2V communications with relays, vehicles are important nodes to forward required data with a receiver and a transmitter at the front and rear, respectively. Each node has two roles in the newtork: one is an Ego vehicle performing cooperative perception with the received information from the previous node, and the other is a relay node simply forwarding the information to the next node. Such receiving and relaying actions are the basic mechanism in V2V communications with relays. In relay networks, the E2E throughput is the minimum cut of link throughputs as $\Gamma_{n}=\min\left\{\gamma_{j}\right\},j\in \left\{1,\cdots ,n\right\}$, where $\Gamma_{n}$ represents the E2E throughput between the *n*-th vehicle and the Detecting vehicle, and $\gamma_{j}$ is the throughput of the *j*-th V2V link. In addition, the throughput of a single V2V link depends on the inter-vehicle distance in the assumed scenarios.

It is necessary to apply the rate control (down sampling) to each node based on the required data rate. As stated in Section 2.2, the required data rate is proportional to the Detecting-Oncoming vehicle distance and inversely proportional to the Ego-Detecting vehicle distance. This strategy can save the spectrum usage and is quite reasonable for the relay-based V2V cooperative perception where transmission capacity diminishes with hop counts.

### 2.4. Numerical Examples

The analysis of required data rate for extended sensors is based on MATLAB. Existing simulators of automated driving systems such as CARLA [19], LGSVL (recently renamed as SVL) [20], have versatile sensing equipment including LiDAR and detection functions. However, they do not support flexible adjustment of LiDAR parameters, especially the angular resolution of LiDAR, because their LiDAR models are built on commercial products. In this study, it is necessary to adapt the angular resolution of LiDAR to the cooperative perception requirements of different Ego vehicles. To this end, we create a geometric model of LiDAR and build a model of the vehicle’s surface composed of dense points according to the actual vehicle parameters (listed in Table 1) in MATLAB. Simulation analysis is implemented with the parameters listed in Table 1. The Field-Of-View (FOV) in vertical and horizontal planes are ±15 degrees and 360 degrees based on the spec of Velodyne LiDAR VLP-16 [21]. In this simulation, the FOV in horizontal plane is assumed to be 180 degrees since the LiDAR only needs to detect the obstacle ahead in overtaking traffic scenarios.

**Figure 4:**
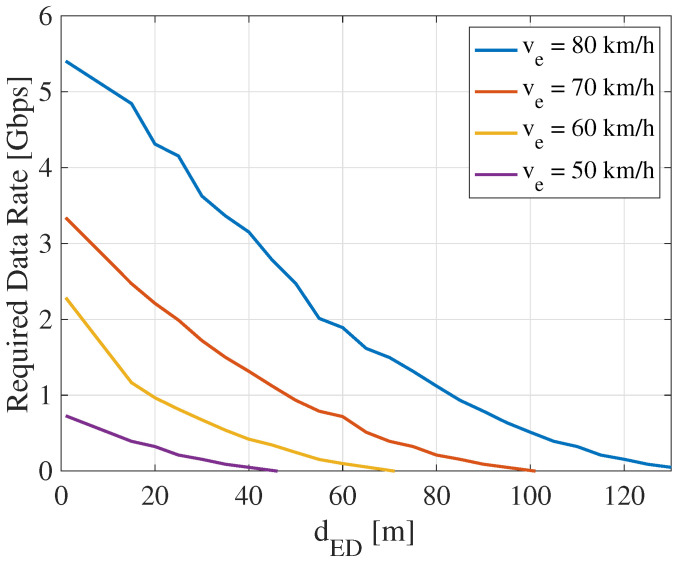
Required data rate of mmWave V2V relays ($d_{\mathrm{ED}}\in \left(0,130\right]$ m, $v_{e}\in \left[50,80\right]$ km/h).

Figure 4 shows the required data rate of V2V relay communications with various $v_{e}$ and $d_{\mathrm{ED}}$. For each vehicle speed $v_{e}$, the required data rate will decrease as the Ego-Detecting vehicle distance $d_{\mathrm{ED}}$ increases. For instance, when $v_{e}$ is 80 km/h (blue line in Figure 4), the required data rate decreases from 5.403 Gbps to 0.049 Gbps as $d_{\mathrm{ED}}$ increases from 1 m to 130 m. For each $d_{\mathrm{ED}}$, by increasing the vehicle speed, the required data rate also grows. When $d_{\mathrm{ED}}$ is equal to 20 m, the required data rate is increased from 0.323 Gbps to 4.31 Gbps as the vehicle speed rises from 50 km/h to 80 km/h. These tendencies of the required data rate are referred to as the basis of resource management.

## 3. Proposal: Distributed Radio Resource Management

### 3.1. Basic Concept

**Figure 5:**
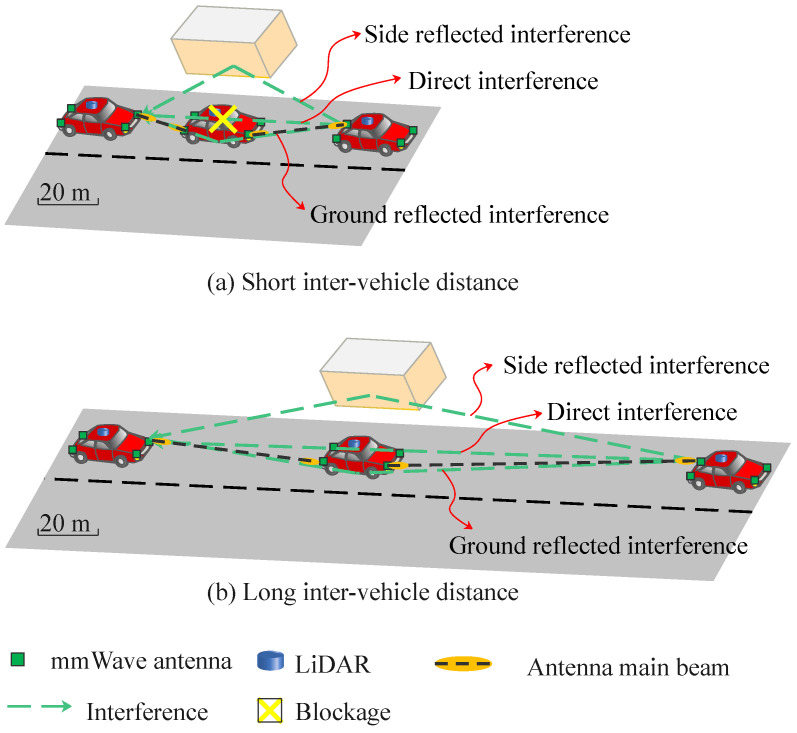
Diagram of ZigZag antenna configuration in short and long inter-vehicle distance scenarios.

MmWave communication links have weak penetration, and they are easily blocked by surrounding obstacles. It is a well-known drawback and barrier to the development of mmWave communications. Meanwhile, this feature can be leveraged for co-channel interference mitigation and spectrum reuse. A single mmWave channel can potentially be used by all V2V links in V2V communication with relays. Our prior work has conceived ZigZag antenna configuration to mitigate the co-channel interference [13]. The scenario in [13] has two features, as drawn in Figure 5a: (1) two mmWave V2V links are connected at the same time by reusing a single mmWave channel, (2) it merely evaluates mmWave V2V performance with short inter-vehicle distance from 10 m to 30 m. In this context, the direct interference can be completely blocked by the front vehicle from the field of view of the third vehicle. Besides, the reflected interference paths from the ground and side objects are effectively suppressed thanks to the ZigZag antenna configuration. The intrinsic principle is to increase the angle between each reflected interference path and the antenna main beam. This increased angle can reduce the antenna directivity of each reflected interference path. Eventually, the total received power of interference can be reduced.

However, in the case of long inter-vehicle distance as depicted in Figure 5b, the direct interference may not be entirely blocked by the front vehicle. Moreover, it is difficult to avoid the reflected interference even by the ZigZag antenna configuration. The angle between each reflected interference path and the antenna main beam becomes extremely small at a long inter-vehicle distance. Therefore, the reflected interference is received with almost the same power as the desired signal. To satisfy the required data rate in the case of long inter-vehicle distance, interference mitigation again becomes a critical challenge.

To cope with this problem, this paper proposes a distributed resource management scheme. The whole bandwidth is divided into two RBs and allocated to each link based on the inter-vehicle distance. The required data rate decreases with the increase of inter-vehicle distance under the same vehicle speed. This is because the Ego-Detecting vehicle distance, $d_{\mathrm{ED}}$, is also lengthened. In the case of short inter-vehicle distance, all RBs are allocated to support the high required data rate for each V2V link. ZigZag antenna configuration is deployed to mitigate the interference when all RBs are reused. On the other hand, in the case of long inter-vehicle distance, divided RBs are alternately allocated to each V2V link in a ZigZag manner which can avoid inter-link interference. If inter-vehicle distances are equal, RB allocation in a ZigZag manner can avoid interference; especially, the interference caused by ground reflection can be completely eliminated. Otherwise, parameters such as the transmit power of each RB should be controlled to mitigate the interference at an arbitrary inter-vehicle distance.

Combining resource management in spatial (ZigZag antenna configuration), frequency (resource block allocation), and power (transmit power control) domains can reduce the overall interference independent of the inter-vehicle distance. It can enhance the achievable E2E throughput in mmWave V2V relay link, which can always satisfy the required data rate.

### 3.2. Definition of Two Modes

**Figure 6:**
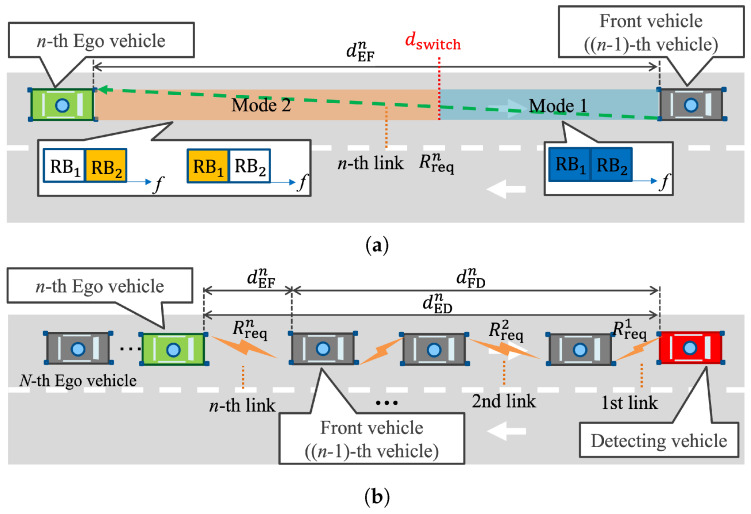
The schematic diagram of mmWave V2V with relays. (**a**) Regions corresponding to two transmission modes. (**b**) The mmWave V2V topology.

Based on the basic concept, two transmission modes are defined to support all V2V links. Figure 6a shows the definition of two transmission modes determined by the inter-vehicle distance. In this figure, the *n*-th Ego vehicle requests the raw data from the (n−1)-th Front vehicle. $d_{\mathrm{EF}}^{n}$ is the straight-line distance between the front of the Ego vehicle and the rear of the Front vehicle, and their communication link is represented as the *n*-th link. $R_{\mathrm{req}}^{n}$ is the required data rate of the *n*-th Ego vehicle. The proposed scheme aims to ensure that the achievable E2E throughput of the *n*-th vehicle ($\Gamma_{n}$) is always higher than $R_{\mathrm{req}}^{n}$. Antennas are installed on the four corners of each vehicle, as shown in the figure. Front antennas are receivers, and rear antennas are transmitters. This antenna installation is used to deploy ZigZag antenna configuration to mitigate interference, especially in the case of short inter-vehicle distance. Then, the inter-vehicle distance can be divided into two regions (colored blue and orange). The transmission modes are designed for each region called Modes 1 and 2. Let $d_{\mathrm{switch}}$ denote the threshold of switching between Modes 1 and 2, which will be optimized based on the required data rate. As a prerequisite, the maximum supported throughput with a full-bandwidth allocation should be higher than the maximum required data rate. For example, 5.403 Gbps is the maximum required data rate at 80 km/h in the overtaking traffic scenarios according to Figure 4. It can be satisfied by full-bandwidth allocation as it is under the upper-bound data rate, i.e., 6.75 Gbps. The whole bandwidth is divided into two RBs in the frequency domain, $\{{\mathrm{RB}}_{1}$ and ${\mathrm{RB}}_{2}\}$, respectively.

When $d_{\mathrm{EF}}^{n}<d_{\mathrm{switch}}$, the transmission mode of the *n*-th link adopts Mode 1. Full bandwidth must be allocated to the current V2V link to meet that high required data rate. When $d_{\mathrm{EF}}^{n}\geq d_{\mathrm{switch}}$, the transmission mode of the *n*-th link adopts Mode 2. A half of full bandwidth is sufficient because its achievable throughput is already higher than the required data rate in this region. When multiple continuous V2V links are in Mode 2, the allocation of ${\mathrm{RB}}_{1}$ and ${\mathrm{RB}}_{2}$ is alternated to avoid the interference.

The transmit powers in Modes 1 and 2 are denoted as $p_{\mathrm{Mode}1}$ and $p_{\mathrm{Mode}2}$, respectively. They will be optimized to guarantee the E2E throughput to satisfy the required data rate when Modes 1 and 2 are alternating in continuous V2V links.

### 3.3. Distributed Radio Resource Management Algorithm

Figure 6b illustrates the mmWave V2V topology. The *n*-th Ego vehicle requests resource management for the *n*-th link to the (n−1)-th Front vehicle. The Front vehicle only needs to know the following local information to complete the resource management for the (n−1)-th link: (1) $d_{\mathrm{EF}}^{n}$, (2) the resource block allocation of the (n−1)-th link, and (3) the working antenna of the (n−1)-th link.

If $d_{\mathrm{EF}}^{n}<d_{\mathrm{switch}}$, all bandwidth is allocated to the *n*-th link by the Front vehicle since the Ego vehicle is driving in the region of Mode 1. The transmit power of the *n*-th link is $p_{\mathrm{Mode}1}$. If $d_{\mathrm{EF}}^{n}\geq d_{\mathrm{switch}}$, ${\mathrm{RB}}_{1}$ or ${\mathrm{RB}}_{2}$ is assigned to the *n*-th link, according to the following condition. If the *n*-th link is the first V2V link (n=1), ${\mathrm{RB}}_{1}$ is allocated to the *n*-th link. When n>1, if the (n−1)-th link uses all bandwidth or ${\mathrm{RB}}_{2}$, ${\mathrm{RB}}_{1}$ is allocated to the *n*-th link. If the (n−1)-th link uses ${\mathrm{RB}}_{1}$, ${\mathrm{RB}}_{2}$ is allocated to the *n*-th link. The detailed workflow is summarized in Algorithm 1. Based on this algorithm, the RB allocation of the *n*-th V2V link, and the transmit power of the *n*-th V2V link, $P_{n}$, can be derived and allocated to the *n*-th link by the (n−1)-th Front vehicle.

As for the ZigZag antenna allocation of the *n*-th link, if the left receiver on the Front vehicle is working for the previous (n−1)-th link, the left transmitter on the Front vehicle is employed for the *n*-th link, and vice versa.

### 3.4. Parameters Optimization

With the randomness of inter-vehicle distance, the continuous two V2V links have four combinations of transmission modes: Mode 1-Mode 1, Mode 1-Mode 2, Mode 2-Mode 1, and Mode 2-Mode 2. In the case of Mode 1-Mode 1, the ZigZag antenna configuration can mitigate the interference. In the case of Mode 2-Mode 2, resource block allocation in the ZigZag manner can be used to avoid interference. In the case of Mode 1-Mode 2 and Mode 2-Mode 1, ZigZag in spatial and frequency domains cannot reduce the impact of interference effectively, so power control is required to handle such a problem. The objective is to find the minimum required transmit power of two transmission modes. In the case of Mode 1-Mode 2 and Mode 2-Mode 1, the minimum required transmit power can ensure that the E2E throughput is always higher than the required data rate. On the other hand, the minimum required transmit power can minimize the interference to other mmWave V2V links. The problem is formulated as

(5) $$\begin{array}{cc} \Omega^{*}= & \arg\min(p_{\mathrm{Mode}1}+p_{\mathrm{Mode}2})\\ \; & s.t.\;\Gamma_{n}\geq R_{\mathrm{req}}^{n},1\leq n\leq N\\ \; & G_{t},G_{r}\leq G_{\max}\\ \; & p_{\mathrm{Mode}1},p_{\mathrm{Mode}2}\leq p_{\max} \end{array}$$

where Ω is a finite set including combinations of parameters. $\Omega^{*}=\left\{p_{\mathrm{Mode}1}^{*},p_{\mathrm{Mode}2}^{*},G_{t}^{*},G_{r}^{*},d_{\mathrm{switch}}^{*},B^{*}\right\}$ represents the optimized parameter values. $G_{t}$ and $G_{r}$ are the antenna gains on the transmitter and receiver, respectively. *B* is the full bandwidth. $p_{\mathrm{Mode}1},p_{\mathrm{Mode}2},G_{t},$$G_{r},d_{\mathrm{switch}},B$ are the parameters to be optimized. $G_{\max},p_{\max}$ represent the constraints, e.g., antenna gain of 47 dBi and transmit power of 10 dBm in the case of 60 GHz band [22].
 **Algorithm 1:** Distributed Resource Block Allocation Scheme for V2V with relays  **Require:** $d_{\mathrm{switch}},\;p_{\mathrm{Mode}1},\;p_{\mathrm{Mode}2},\;d_{\mathrm{EF}}^{n}$  **Ensure:** Resourceblockallocationofn-thlink     $\mathrm{Allocated}\;\mathrm{transmit}\;\mathrm{power}\;\mathrm{of}\;n-\mathrm{th}\;\mathrm{link}\;:\;P_{n}$    **if**
$\;d_{\mathrm{EF}}^{n}<d_{\mathrm{switch}}$
**then**     n-thlinkbelongstoMode1.     n-thlink←Allbandwidth     $P_{n}\leftarrow p_{\mathrm{Mode}1}$    **else**     n-thlinkbelongstoMode2.     $P_{n}\leftarrow p_{\mathrm{Mode}2}$     **if** n=1 **then**      $n-\mathrm{th}\;\mathrm{link}\;\leftarrow {\mathrm{RB}}_{1}$     **else**      **if** (n-1)-thlink←Mode1      $\mathrm{or}\;\left(n-1\right)-\mathrm{th}\;\mathrm{link}\;\leftarrow \;\mathrm{Mode}\;2\;{\mathrm{RB}}_{2}$
**then**       $n-\mathrm{th}\;\mathrm{link}\;\leftarrow {\mathrm{RB}}_{1}$      **else**       $n-\mathrm{th}\;\mathrm{link}\;\leftarrow {\mathrm{RB}}_{2}$      **end if**     **end if**    **end if**    **return**$\;\mathrm{RB}\;\mathrm{allocation}\;\mathrm{result}\;\mathrm{of}\;n-\mathrm{th}\;\mathrm{link}\;,\;P_{n}$

## 4. Performance Evaluation

Now, IEEE 802.11bd, as a successor of IEEE 802.11p, is being specified for next generation V2X (NGV) communications. Besides the 5.9 GHz, IEEE 802.11bd also targets the mmWave frequency band at 57–71 GHz [5]. In this target frequency band, 63–64 GHz is allocated to ITS in the Europe [23], and there is an ongoing work in ITU-R to allocate 60 GHz band for ITS. In the PHY layer, IEEE 802.11bd is assumed to utilize IEEE 802.11ad. Therefore, here we verify the effectiveness of the proposed scheme at 60 GHz (IEEE 802.11ad) in the PHY layer for mmWave V2V relay communications. In the MAC layer, IEEE 802.11bd borrows the rules of IEEE 802.11p to reduce the association overhead. For example, the Outside of the Context of BSS (OCB) rules allow vehicles to transmit signals without prior association [24]. The evaluation of the proposed distributed and scalable radio resource management scheme is also implemented in MATLAB. The most commonly used road traffic simulator, SUMO [25], has been integrated into Veins to provide vehicular connectivity [26]. However, it only supports IEEE 802.11p-based V2X communications, lacking mmWave communications and ray-tracing channel models. In this study, establishing the ray-tracing channel model is necessary to analyze the co-channel inter-link interference. Therefore, we do ray-tracing-based channel modeling at 60 GHz in MATLAB to derive the received power of desired signal and co-channel inter-link interference. First, the optimized parameters of the two transmission modes with different antenna specs are analyzed. Then, the achievable E2E throughput under four situations is compared with the required data rate, which demonstrates the effectiveness of our proposed resource control scheme.

### 4.1. Simulation Assumptions

Before showing the simulation result, the following assumptions are emphasized. Detailed parameters are listed in Table 2.

- Antennas are located at the center of front and rear of the vehicle under the conventional antenna configuration;
- The transmit power is uniformly distributed to each RB;
- Both ground reflection and surrounding reflection are considered as the main interference;
- The standard deviation of vertical fluctuation caused by the motors of vehicles is set to 0.0319 m [27] to evaluate the beam alignment error due to the narrow beam width;
- The antenna gains on the transmitter and receiver sides are assumed to be the same and a general radiation pattern of rectangular aperture antenna is used in this simulation as $G_{t}=G_{r}=\frac{4\pi }{\frac{\pi \theta_{\mathrm{HP}}^{\circ }}{180}\frac{\pi \phi_{\mathrm{HP}}^{\circ }}{180}}$, where $\theta_{\mathrm{HP}}^{\circ }$ and $\phi_{\mathrm{HP}}^{\circ }$ are the beam width of antennas in the vertical and horizontal planes respectively [28];
- To support the RB allocation, the simulation is analyzed based on Orthogonal Frequency Division Multiplex PHY (OFDMPHY). The supported MCS (Modulation and Coding Scheme) index is 13–24;
- Channel gain of each RB is determined based on the ray-tracing model assuming urban street canyon scenario, which considers ground and wall reflections [13];
- The Signal-to-Interference-plus-Noise Ratio (SINR) of ${\mathrm{RB}}_{m},m\in \left\{1,2\right\}$ for the *n*-th link can be calculated by
  (6) $$\beta_{nm}=\frac{r_{nm}}{I_{nm}+\frac{p_{\mathrm{noise}}}{n_{\mathrm{RB}}}},$$
  where $r_{nm}$ is the received power of ${\mathrm{RB}}_{m}$ for the *n*-th link, $I_{nm}$ is the interference of ${\mathrm{RB}}_{m}$ for the *n*-th link when the previous links also reuse the same RB. $p_{\mathrm{noise}}$ is the power of Additive White Gaussian Noise (AWGN) for all RBs and $n_{\mathrm{RB}}$ is the number of RBs;
- The throughput of the *n*-th link depends on $\beta_{nm}$ and is determined by referring to MCS table;
- The E2E throughput between the *n*-th Ego vehicle and the Detecting vehicle is the minimum cut of link throughput as $\Gamma_{n}=\min\left\{\gamma_{j}\right\},j\in \left\{1,\cdots ,n\right\}$.

### 4.2. Results: Parameters Optimization

First, the parameters are optimized based on Equation (5). Here, the required data rate at 80 km/h is selected as a basis of radio resource management. The proposed scheme is still applicable to other vehicle speeds.

According to the analysis of [13], the worst interference case is on a single straight road with equal inter-vehicle distance. Therefore, this paper compares the achievable E2E throughput of Modes 1 and 2 with the required data rate in the worst case to determine the value of $d_{\mathrm{switch}}$. If the optimized $d_{\mathrm{switch}}$ can support the E2E throughput larger than the required data rate in the worst case, the optimized $d_{\mathrm{switch}}$ can also help other situations. It should be noted that the throughput of each mmWave V2V link is maintained or reduced due to the effect of interference as the hop count increases under the equal inter-vehicle distance. This means that the E2E throughput from each Ego vehicle to the Detecting vehicle is equal to the throughput between the Ego vehicle and its Front vehicle as $\Gamma_{n}=\min\left\{\gamma_{j}\right\}=\gamma_{n},j\in \left\{1,\cdots ,n\right\}$ in the situation of equal inter-vehicle distance.

In IEEE 802.11ad, the maximum achievable throughput of a single channel with 2.16 GHz bandwidth at 60 GHz carrier frequency is 6.757 Gbps. To support the maximum required data rate of 5.403 Gbps at 80 km/h, the minimum required full bandwidth can actually be lower than 2.16 GHz. Considering the practical use of this resource management scheme in 60 GHz and facilitating the deployment by fitting the commercialized IEEE 802.11ad standard, the value of 2.16 GHz is kept as the minimum required full bandwidth in this simulation.

Table 3 shows the minimum required transmit power of two modes under the different antenna specifications. The range of antenna gain is set to [30, 36] dBi. If the antenna gain is less than 30 dBi, the wider antenna beam width hinders the effectiveness of interference suppression using the ZigZag antenna configuration. If the antenna gain is greater than 36 dBi, a too narrow beam width reduces the tolerance to vehicle vibration, which causes severe beam alignment errors. Only the antenna gain in this range can ensure that the E2E throughput satisfies the required data rate. Therefore, under different antenna specifications within the range of antenna gain, the corresponding minimum required transmit power of two modes can be found by the optimization.

**Figure 7:**
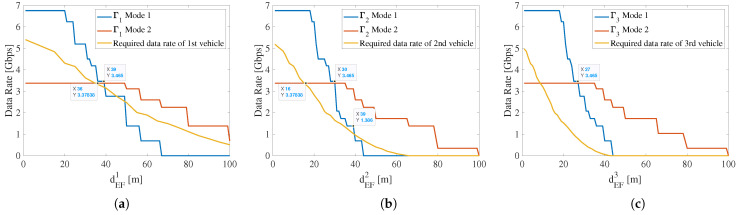
Comparison between achievable E2E throughput (Modes 1 and 2) and required data rate ($G_{t}=G_{r}=30\;\mathrm{dBi},\;p_{\mathrm{Mode}1}=-11\;\mathrm{dBm},\;p_{\mathrm{Mode}2}=4\;\mathrm{dBm},\;B=2.16\;\mathrm{GHz},\;d_{\mathrm{EF}}^{n}\in \left(0,100\right]$). (**a**) First vehicle; (**b**) second vehicle; (**c**) third vehicle.

To determine the optimized $d_{\mathrm{switch}}$, the E2E throughput of Modes 1 and Mode 2 are compared with the required data rate as shown in Figure 7. Because the first V2V link cannot suffer from strong interference from the front, it is regarded as a particular case. In the distributed resource allocation mechanism, it is easy to identify the first V2V link. Therefore, the parameters such as $d_{\mathrm{switch}}$ of the first V2V link are different from other V2V links based on practical applications. Figure 7a shows the comparison between E2E throughput (Modes 1 and 2) and the required data rate in the particular case. In this figure, when $d_{\mathrm{EF}}^{1}>39$ m, $\Gamma_{1}$ in Mode 1 is lower than the required data rate of the first vehicle because of the severe path loss and multipath fading. However, when $d_{\mathrm{EF}}^{1}>36$ m, $\Gamma_{1}$ in Mode 2 is higher than the required data rate since the higher transmit power of Mode 2 can compensate for the increased path loss in long inter-vehicle distance. Therefore, for the first link, the possible range of $d_{\mathrm{switch}}$ is [36,39] m. There should be a reserved margin between E2E throughput and the required data rate, especially for the first link. This margin is critical since the practical E2E throughput may not reach the theoretical value due to the hardware limitations and other impact factors such as congestion control and packet loss. To this end, $p_{\mathrm{Mode}1}$ and $p_{\mathrm{Mode}2}$ are set to be higher than the minimum required transmit power for algorithm robustness.

In Figure 7b,c, the distance values corresponding to the intersections are 30 m and 27 m, which are smaller than those in Figure 7a (39 m). This is because the second link to the *N*-th link suffers from the interference caused by its previous links. These interferences make the $\Gamma_{2}$ Mode 1 and $\Gamma_{3}$ Mode 1 reduce more rapidly than $\Gamma_{1}$ Mode 1. In Figure 7b, since the required data rate of the second vehicle decreases, the range of $d_{\mathrm{switch}}$ for the second link is expanded to [16,39] m. As for the third link, both Modes 1 and 2 can support $\Gamma_{3}$ to always keep more than the required data rate for the third vehicle. There is no limitation on the value of $d_{\mathrm{switch}}$. Although the range of $d_{\mathrm{switch}}$ becomes wider, the optimized $d_{\mathrm{switch}}$ better be 30 m or 27 m to reserve the margin for possible E2E throughput degradation in the actual deployment. To ensure the scalability of this radio resource management scheme, the value of $d_{\mathrm{switch}}$ should be independent of the V2V link index. Therefore, the optimized range of $d_{\mathrm{switch}}$ applicable for the first link can be determined as [36,39] m and the optimized $d_{\mathrm{switch}}$ for the second link to the *N*-th link is about 30 m.

### 4.3. Results: Achievable E2E Throughput

**Figure 8:**
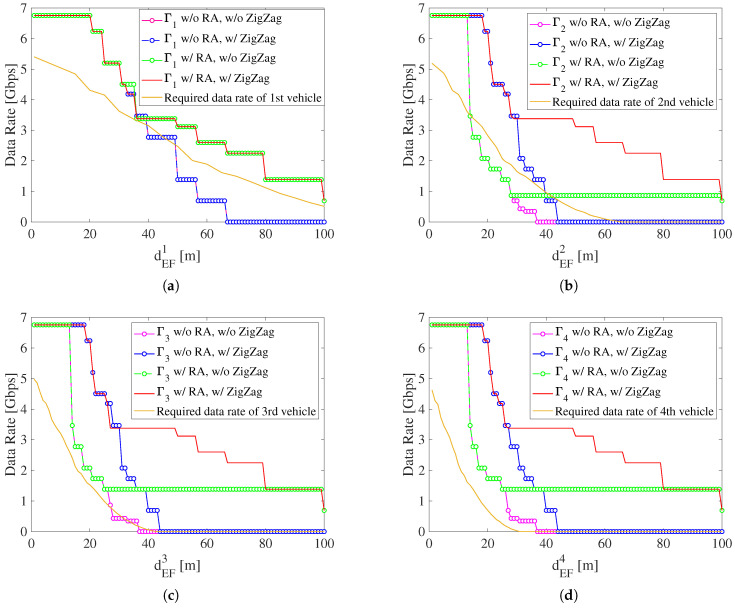
Comparison between achievable E2E throughput and required data rate with equal inter-vehicle distance ($G_{t}=G_{r}=30\;\mathrm{dBi},\;p_{\mathrm{Mode}1}=-11\;\mathrm{dBm},\;p_{\mathrm{Mode}2}=4\;\mathrm{dBm},\;d_{\mathrm{switch}}=36\;m$(n=1)or30m$\left(n>1\right),\;B=2.16\;\mathrm{GHz},\;d_{\mathrm{EF}}^{n}\in \left(0,100\right]$). (**a**) First vehicle; (**b**) second vehicle; (**c**) third vehicle; (**d**) fourth vehicle.

Figure 8 presents the comparison between achievable E2E throughput in the worst case under four situations and the required data rate. The four situations include: (1) w/o resource block allocation (RA), w/o ZigZag antenna configuration; (2) w/o RA, w/ ZigZag antenna configuration; (3) w/ RA, w/o ZigZag antenna configuration; (4) w/ RA, w/ ZigZag antenna configuration. The situation of w/o RA means that the V2V link keeps Mode 1. The w/o ZigZag antenna configuration situation means that antennas are located at the center of a vehicle’s front and rear.

From Figure 8a, when $d_{\mathrm{EF}}^{1}\geq 36$ m, $\Gamma_{1}$ w/o RA is lower than the required data rate of the first vehicle because of the strong path loss and multipath fading. $\Gamma_{1}$ w/ RA is always higher than the required data rate since Mode 1 is switched to Mode 2 when $d_{\mathrm{EF}}^{1}\geq 36$ m. The higher transmit power in Mode 2 can compensate for the high path loss. In addition, ZigZag antenna configuration does not affect $\Gamma_{1}$ because the first link does not suffer from the interference.

From Figure 8b, when $d_{\mathrm{EF}}^{2}\in \left[18,65\right]$ m, $\Gamma_{2}$ w/o RA and w/o ZigZag is lower than the required data rate of the second vehicle because second link V2V suffers from the strong interference. To cope with this problem, when $d_{\mathrm{EF}}^{2}\in \left[18,30\right)$ m, due to the short inter-vehicle distance, the ZigZag antenna configuration is used to suppress the strong interference. When $d_{\mathrm{EF}}^{2}\in \left[30,65\right]$ m, the interference suppression by only using ZigZag antenna configuration becomes weak because of long inter-vehicle distance, so the resource block allocation is used to avoid the interference. Since ${\mathrm{RB}}_{1}$ and ${\mathrm{RB}}_{2}$ are allocated to the first and second links respectively, the interference especially ground reflection can be eliminated. Therefore, $\Gamma_{2}$ w/ RA and ZigZag antenna configuration can always keep more than the required data rate of the second vehicle.

From Figure 8c, the curves of $\Gamma_{3}$ with four situations are almost the same as those of $\Gamma_{2}$, which means that the first link before the current link causes serious interference, and the interference caused by other earlier links can be neglected. Here, increasing the index of the V2V link reduces the required data rate at the same $d_{\mathrm{EF}}$ because the required data rate is a function of $d_{\mathrm{ED}}^{n}$. For the third link, only when $d_{\mathrm{EF}}^{3}\in \left[30,42\right]$ m, $\Gamma_{3}$ w/o RA is lower than the required data rate of the third vehicle. In Figure 8d, when the V2V link index is 4, $\Gamma_{4}$ is always higher than the required data rate of the fourth vehicle regardless of RA and ZigZag antenna configuration. Therefore, when n>4, the achievable E2E throughput also surpasses the required data rate. It can be concluded that the proposed distributed radio resource management scheme is independent of the V2V link index, which implies that this scheme can support scalable mmWave V2V communications topology.

**Figure 9:**
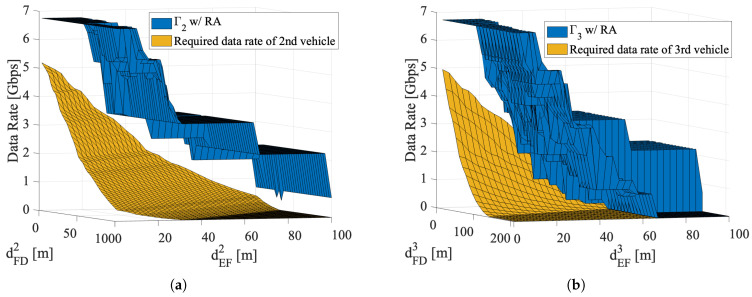
Comparison between achievable E2E throughput and required data rate with arbitrary inter-vehicle distance ($G_{t}=G_{r}=30\;\mathrm{dBi},\;p_{\mathrm{Mode}1}=-11\;\mathrm{dBm},\;p_{\mathrm{Mode}2}=4\;\mathrm{dBm},\;d_{\mathrm{switch}}=36\;m$(n=1) or 30m$\;(n>1),\;B=2.16\;\mathrm{GHz},\;d_{\mathrm{EF}}^{n}\in \left(0,100\right]$). (**a**) Second vehicle; (**b**) third vehicle.

Figure 9 compares the achievable E2E throughput with the required data rate at arbitrary inter-vehicle distances. In the situation of arbitrary inter-vehicle distance, $\Gamma_{n}=\min\left\{\gamma_{j}\right\}$. In Figure 9a, $\Gamma_{2}$ provided by the proposed scheme can ensure the required data rate irrespective of both $d_{\mathrm{FD}}^{2}$ and $d_{\mathrm{EF}}^{2}$ from 1 m to 100 m. In Figure 9b, $\Gamma_{3}$ can also keep more than the required data rate of the third vehicle regardless of variation of $d_{\mathrm{FD}}^{3}$. Therefore, the proposed distributed radio resource management scheme is independent with $d_{\mathrm{FD}}^{n}$.

The above results verify the effectiveness of the proposed scheme that can achieve sufficient E2E throughput performance to ensure safe automated driving.

## 5. Conclusions

In this paper, the theoretical analysis of the required data rate for safe automated driving was given in an overtaking scenario with multiple Ego vehicles. The simulation results revealed that the increase of Ego vehicles alleviates their required data rate. With these preparations, a novel distributed and scalable radio resource management scheme was proposed for mmWave V2V relay communication. It optimizes the allocation of resource blocks in spatial, frequency, and power domains. The simulation results demonstrated that our proposal ensures that the achievable E2E throughput is always higher than the required data rate for each vehicle. It also verifies the scalability of the proposed scheme for dynamics of mmWave V2V communication topology. It can be a fundamental strategy for the realization of the coming automated driving era.

## Figures and Tables

**Table 1:** Simulation parameters for LiDAR detection.

Parameters	Value
Width of lane ($w_{\mathrm{lane}}$)	3.2 m
Length of vehicle ($l_{\mathrm{vehicle}}$)	4.35 m
Width of vehicle ($w_{\mathrm{vehicle}}$)	1.69 m
Height of vehicle ($h_{\mathrm{vehicle}}$)	1.48 m
Height of metal body of vehicle ($h_{\mathrm{mb}}$)	1.1 m
Chassis clearance	0.14 m
Height of LiDAR ($h_{l}$)	1.6 m
FOV (vertical)	30 degrees
	(±15 degrees)
FOV (horizontal)	180 degrees
Scan frequency (*f*)	20 Hz
Number of bits per laser ($B_{\mathrm{laser}}$)	28 bits
Number of points of vehicle’s surface ($N_{\mathrm{vehicle}}$)	2615

**Table 2:** Simulation parameters for V2V relay communications.

Parameters	Value
Number of RBs ($n_{\mathrm{RB}}$)	2
Carrier frequency (*f*)	60 GHz
Height of antenna (*h*)	1 m
MCS index	13–24
Dielectric constant of asphalt	3.9975-j0.2
Dielectric constant of concrete	4.94-j0.69
Noise figure (NF)	10 dB
Noise power density ($N_{0}$)	−174 dBm/Hz
Speed of Ego vehicle ($v_{e}$)	80 km/h
Distance between right	1.69 m
and left antennas ($d_{a}$)	
Variation of inter-vehicle distance	(0,100] m

**Table 3:** Minimum required transmit power of two modes.

$G_{t}=G_{r}$ (dBi)	$p_{\mathrm{Mode}1}$ (dBm)	$p_{\mathrm{Mode}2}$ (dBm)
36	−25	−15
34	−20	−7
32	−15	−1
30	−11	4

## Data Availability

Not applicable.

## Abbreviations

The following abbreviations are used in this manuscript:

mmWave	Millimeter-wave
V2V	Vehicle-to-Vehicle
V2X	Vehicle-to-Everything
E2E	End-to-End
NR-V2X	New Radio V2X
3GPP	3rd-Generation Partnership Project
LTE	Long Term Evolution
CSMA/CA	Carrier Sense Multiple Access with Collision Avoidance
RBs	Resource Blocks
LiDAR	Light Detection And Ranging
FOV	Field-Of-View
OFDMPHY	Orthogonal Frequency Division Multiplex PHY
MCS	Modulation and Coding Scheme
SINR	Signal-to-Interference-plus-Noise Ratio
AWGN	Additive White Gaussian Noise
RA	Resource Block Allocation
